# Childhood Maltreatment, Bullying, and Internet Addiction in Relation to Suicidal Ideation Among Adolescents: Cross-Sectional Mediation and Network Analysis

**DOI:** 10.2196/79858

**Published:** 2025-10-07

**Authors:** Jiayi Lu, Sihong Li, Tianqing Fan, Xi Ni, Leyin Zhang, Hui Chen, Xianliang Chen, Huajia Tang, Yanyue Ye, Jiansong Zhou, Yanmei Shen

**Affiliations:** 1 Department of Psychiatry, National Clinical Research Center for Mental Disorders, and National Center for Mental Disorders, The Second Xiangya Hospital of Central South University, 139 Renmin Middle Road, Furong DistrictChangsha, 410011, China, 86 13170401359

**Keywords:** suicide ideation, internet addiction, bullying, childhood maltreatment, network analysis, mediation analysis

## Abstract

**Background:**

Internet addiction (IA), childhood maltreatment (CM), and bullying are prevalent psychosocial stressors among adolescents and have each been associated with suicidal ideation (SI). However, existing research often treats these factors in isolation, overlooking their potential interrelationships and joint associations with SI.

**Objective:**

This study aimed to examine how CM, IA, and bullying are jointly related to SI at both the scale and symptom levels and identify key symptoms within the CM-IA-bullying-SI network that may serve as intervention targets to disrupt maladaptive associations across the network.

**Methods:**

A total of 6573 adolescents were recruited through cluster sampling. Mediation analyses were conducted to assess direct and indirect effects of CM on SI via IA and bullying. Network analysis was conducted to examine symptom-level associations among CM, IA, bullying, and SI and identify core and bridge symptoms within the network. Network comparison tests were conducted to assess differences in network structure by gender and history of nonsuicidal self-injury.

**Results:**

Mediation analyses revealed that both IA and bullying partially mediated the association between CM and SI, with significant indirect effects via IA (*c’*=0.010, 95% CI 0.008-0.011; *P*<.001) and bullying (*c’*=0.004, 95% CI 0.002-0.005; *P*<.001). In the network, *tolerance*, *time management*, and *compulsive internet use* were identified as central symptoms, whereas *SI*, *emotional abuse*, and *traditional bullying victimization* served as bridge symptoms. *Emotional abuse* and *cyberbullying victimization* were most strongly linked to SI. Among individuals with a history of nonsuicidal self-injury, *emotional abuse* and *emotional neglect* showed stronger associations with SI. Sex subgroup analysis showed no significant difference in global strength (*S*=0.095; *P*=.69) but a significant difference in network structure (*M*=0.174; *P*=.01).

**Conclusions:**

This study revealed how CM, bullying, and IA are jointly related to SI among adolescents at both the scale and symptom levels. Key symptoms, including tolerance and time management, played central roles within the symptom network, with SI bridging multiple psychosocial domains. These findings underscore the need for multilevel, targeted interventions to disrupt maladaptive links and reduce suicide risk in adolescents.

## Introduction

### Background

Adolescence is a critical developmental stage characterized by rapid biological, psychological, and environmental changes, making this period particularly susceptible to mental health challenges [1]. Recent studies have reported a concerning rise in suicidal ideation (SI) among adolescents, with prevalence rates ranging from 14.3% to 22.6% across regions, underscoring a growing public health concern  [2,3]. There is emerging evidence suggesting that this upward trend may be partly driven by evolving environmental stressors such as increased digital exposure, changing patterns of peer interaction, and shifts in family dynamics [4-6]. These developments suggest that the factors associated with SI in today’s youth may differ from those of previous generations and present more complexity [7]. To understand how these diverse influences shape adolescent mental health nowadays, the ecological systems theory offers a useful framework, proposing that individual development is shaped by multiple nested layers of influence, such as family, school, digital environments, and the broader society [8,9]. Given this complexity, it is essential to examine how internet use, peer relationships, and family factors are jointly associated with adolescent SI. Clarifying these associations may not only advance theoretical understanding but also inform more comprehensive and context-sensitive suicide prevention strategies.

In the context of evolving social environments, widespread internet use among adolescents has raised significant concerns. In China, internet penetration among youth has reached over 97%, with more than 160 million users aged 10 to 19 years as of 2023 [10-12]. While the internet offers educational and social opportunities, its immersive nature, combined with adolescents’ developmental vulnerabilities, increases the risk of internet addiction (IA) [13,14]. IA is characterized by excessive and uncontrolled use that leads to psychological, social, and functional impairments, with prevalence among Chinese adolescents estimated at 6.3% to 26.5% [15-17]. Accumulating evidence links IA to a range of adverse outcomes, including emotional dysregulation; academic difficulties; and, most critically, heightened risks of SI and nonsuicidal self-injury (NSSI) [18-20]. Recognizing these severe consequences, the *Diagnostic and Statistical Manual of Mental Disorders, Fifth Edition,* identifies IA as a condition requiring further research [21]. Notably, IA encompasses multiple symptom dimensions, such as impulsiveness, avoidance, and social withdrawal, which overlap with key features of SI [22,23]. These overlapping features suggest a complex, multifaceted association between IA and SI. Therefore, a more nuanced understanding of this relationship is warranted. Examining this association at both the total scale and item-specific symptom levels may help clarify risk pathways and inform more precise prevention strategies for adolescents.

Widespread internet use has also reshaped adolescent peer interactions, contributing to the rise of cyberbullying alongside traditional forms of bullying [24]. Traditional bullying involves repeated aggressive behaviors, whether as a victim or perpetrator, aimed at causing physical, emotional, or social harm [25]. Cyberbullying refers to deliberate and repetitive harmful actions conducted via online platforms [26]. Both forms are linked to adverse mental health outcomes and appear more frequently among adolescents with IA [24,27]. Research suggests a bidirectional relationship between bullying and IA. On the one hand, involvement in bullying increases the risk of IA, particularly in cases of cyberbullying, where anonymity and the larger scale intensify the impact of cyberbullying [27,28]. On the other hand, individuals with IA may be more likely to engage in or be exposed to bullying due to their heightened online activity. On the basis of problem behavior theory, adolescent risk behaviors such as bullying and IA often cluster together due to shared psychological and social mechanisms [29]. Victims may turn to excessive internet use to cope with emotional distress or isolation, whereas perpetrators may use online spaces to assert dominance, reinforcing excessive internet use [30-32]. This interplay suggests that IA and bullying not only are independently associated with SI but may also compound each other’s psychological burden. IA may magnify the emotional impact of bullying (particularly cyberbullying), thereby accelerating the onset or severity of SI [33,34]. These findings highlight the need to examine IA and bullying not in isolation but as interrelated processes that jointly contribute to suicide risk in adolescents.

The family environment plays a fundamental role in adolescent development, particularly given adolescents’ heightened emotional need regarding familial relationships. Among family-related risk factors, childhood maltreatment (CM), including emotional and physical neglect (PN) or abuse, has been consistently associated with elevated risk of SI [35]. CM may be associated with SI through multiple psychological pathways, such as emotional dysregulation, alexithymia, and feelings of powerlessness [36-38]. Importantly, CM rarely occurs in isolation. It frequently coexists with other psychosocial risk factors, particularly IA and bullying. Evidence shows that childhood neglect and abuse significantly increase the likelihood of IA in adolescence, with risk increasing alongside CM severity [39,40]. In parallel, CM has also been associated with both bullying perpetration and bullying victimization; adolescents exposed to maltreatment may be more likely to bully others [41] or, particularly in cases of emotional abuse (EA), become targets of school bullying themselves [42]. The cumulative psychological burden of CM, IA, and bullying, including emotional problems and social withdrawal, may further heighten the risk of severe mental health outcomes, including SI [16,43]. While the individual associations between these factors and SI are well documented, their interrelatedness at the symptom level remains underexplored [16,19,20,33,34,43]. Investigating how CM, IA, and bullying jointly contribute to SI may yield novel insights into shared vulnerabilities. Addressing this gap could inform integrated intervention strategies that simultaneously target multiple psychosocial stressors rather than treating them in isolation.

Furthermore, these associations may differ by gender. There is evidence suggesting that male individuals are more susceptible to IA, whereas female individuals show higher levels of SI [44,45]. Gender differences have also been observed in bullying involvement and the psychological effects of CM [46,47]. In addition, SI frequently co-occurs with NSSI, a related but distinct behavior characterized by deliberate self-harm without suicidal intent [48]. Although SI and NSSI share common mechanisms such as emotional dysregulation, their partial overlap reflects behavioral heterogeneity [49]. Adolescents experiencing NSSI may exhibit more tightly connected symptom networks, suggesting greater vulnerability to interacting risk factors such as IA, CM, and bullying [50]. These findings underscore the need for gender- and NSSI status–stratified analyses to better inform targeted prevention strategies.

### Objectives

Building on the existing literature, this study adopted 2 complementary analytic approaches to investigate the relationships among CM, IA, bullying, and SI in adolescents. First, mediation analysis was used to examine potential indirect pathways linking these psychosocial risk factors to SI. Second, cross-sectional network analysis was conducted to explore symptom-level associations and identify central and bridge symptoms across IA, CM, bullying, and SI domains. To capture potential subgroup heterogeneity, analyses were stratified by gender and NSSI status. This study aimed to address two critical objectives: (1) given that CM typically occurs earlier in life, we explored the associations among CM, IA, bullying, and SI at the total score level to assess potential mediating patterns linking these psychosocial factors; and (2) to achieve a more in-depth understanding, we constructed a network of CM, IA, bullying, and SI symptoms encompassing specific items. We hypothesize that IA and bullying serve as potential mediators in the association between CM and SI. We also hypothesize that, in the network analysis, specific CM, IA, and bullying items and SI will be positively related to each other. This integrative framework enables the simultaneous examination of psychological pathways and observable symptom-level structures underlying suicide risk in adolescents, offering insights that may inform multidimensional prevention strategies.

## Methods

### Study Design, Participants, and Setting

This cross-sectional study used cluster sampling to select participants from 2 middle schools in Changsha, Hunan province, and Chongqing, China, from October 2021 to November 2021. Questionnaires were generated using the online platform Wenjuanxing and distributed via WeChat with the assistance of school staff. All students aged 12 to 17 years were eligible for participation. Exclusion criteria included any adolescents with severe physical or mental health conditions. A total of 6573 valid responses were collected and included in the final analysis, far exceeding typical thresholds for mediation [51] and psychological network analyses, which have been shown to achieve reliable results with as few as 250 to 350 participants when networks are sparse and include fewer than 20 nodes [52]. All participants completed the same standardized questionnaires under consistent instructions from a trained research team.

### Assessments

#### Demographics

A self-designed questionnaire was used to collect the demographic information of the participants, including age, gender, ethnicity, and whether they were an only child.

#### CM Assessment

We used the Childhood Trauma Questionnaire–Short Form to assess CM [53]. This self-report questionnaire consists of 28 items and is divided into 5 subscales: EA, physical abuse, sexual abuse, emotional neglect (EN), and PN. Given that sexual abuse is a sensitive topic, it was excluded from the survey, and the cutoff values for the other 4 subscales were 13 for EA, 10 for physical abuse, 15 for EN, and 10 for PN [54]. Each item was rated on a 5-level Likert scale ranging from 1 (“none”) to 5 (“always”). The Childhood Trauma Questionnaire–Short Form has good psychometric properties [55], and its Cronbach α was 0.81 in this study.

#### IA Assessment

The 19-item revised Chinese Internet Addiction Scale, adapted by Yu and Fu-min [56], was used to assess IA. Participants were asked to answer each question on a 4-point scale ranging from 1 (“very inconsistent”) to 4 (“very consistent”), with higher scores indicating greater IA. The scale consists of 5 dimensions: compulsive internet use (3 items), withdrawal reaction (3 items), tolerance (4 items), interpersonal and health-related problems (5 items), and time management problems (4 items). The score for each dimension is calculated by adding the scores of the items within that dimension. A total score above 53 is considered to be indicative of IA, and a score less than 46 is classified as normal. This scale has good reliability and validity and has been widely used [57]. In our sample, the Chinese Internet Addiction Scale exhibited outstanding reliability, with a Cronbach α of 0.97.

#### Bullying

Bullying was assessed through 4 self-reported items, including both traditional bullying and cyberbullying. The first 2 questions were victim version: “Have you been bullied in the last 12 months?” “In the past 12 months, has anyone used the Internet, text messaging, Weibo, WeChat (or any other electronic device) to bully, tease, or threaten you?” The following 2 questions were the bully version: “Have you bullied anyone in the last 12 months?” “ In the past 12 months, have you used the Internet, text messaging, Weibo, WeChat (or any other electronic device) to bully, tease, or threaten someone?” A 4-point continuous scale was used to score (0=“never,” 1=“once,” 2=“twice,” and 3=“more than twice”). For the mediation analysis, the total bullying score was computed by summing responses to the 4 individual items, with higher scores indicating greater involvement in bullying. The Cronbach α value of this scale was 0.84.

#### SI Assessment

Participants’ SI was assessed using 2 questions. The first question asked the following: “Have you ever experienced suicide ideation?” The second question inquired the following: “How many times have you experienced suicide ideation in the past 12 months?” A 5-point scale was used for scoring (0=“none,” 1=“once,” 2=“twice,” 3=“3‐5 times,” and 4=“more than 5 times”). Participants who reported never having experienced SI were classified as the group without SI, whereas those who reported having SI were classified as the SI group.

#### NSSI Assessment

We assessed NSSI by asking participants whether they had intentionally harmed themselves in the previous year without the intention of ending their lives. This included behaviors such as hair pulling; head banging; and hitting, pinching, scratching, biting, scalding, or cutting themselves. Previous studies have shown that this assessment has good reliability and validity among Chinese adolescents [58]. On the basis of their responses, participants were classified into NSSI and non-NSSI groups.

### Statistical Analyses

#### Descriptive Analyses

This study used frequencies and percentages to report categorical variables and means and SDs to report continuous variables. Given the nonnormal distribution of continuous variables, the chi-square test was used for categorical variables, and the Mann-Whitney *U* test was applied to compare the differences in sociodemographic and psychological characteristics between the SI and non-SI groups. All descriptive analyses were conducted using SPSS (version 26.0; IBM Corp), with a 2-tailed test and a significance level set at *P*<.05.

#### Mediation Analyses

To examine the associations among CM, IA, bullying, and SI, we first conducted 2 simple mediation models using the PROCESS macro (model 4) for SPSS (version 4.1). IA and bullying were tested separately as potential mediators of the relationship between CM (independent variable) and SI (dependent variable). The 5000-bootstrap resampling method was used to estimate the 95% CI. A mediation effect was considered significant if the 95% CI did not include 0.

#### Network Estimation

Network analysis was used to explore the correlations among IA, SI, CM, and bullying at the symptom level. To further examine the potential influence of gender and NSSI on the network, we constructed separate IA-SI-CM-bullying networks for male and female subsamples, as well as for subsamples with and without a history of NSSI. Finally, 5 independent network models were included, and all of them were constructed using the *qgraph* package in R (version 4.3.1; R Foundation for Statistical Computing) [59]. Due to the nonnormal distribution of continuous data, a Gaussian graphical model was constructed based on the Spearman correlation. This is the most commonly used type of undirected network, where a “node” represents a variable and an “edge” represents the strength of the conditional association (partial correlation coefficient) between 2 variables, after controlling for the effects of all other variables in the network [60,61]. Red dashed edges indicate negative correlations, whereas blue edges indicate positive correlations.

In addition, according to recent guidelines [62], the *ggmModSelect* algorithm is reported to perform better with large samples (N>5000); therefore, we used this approach to construct the network model. Specifically, the *ggmModSelect* algorithm begins by applying least absolute shrinkage and selection operator regularization to create several network structures. After that, it removes the regularization from each network and reevaluates the nonzero edges using maximum likelihood estimates. Then, by optimizing the Bayesian information criterion, the accuracy of model selection is further enhanced, allowing for a more effective capture of the true conditional dependence between variables [62,63]. Finally, network visualization uses the Fruchterman-Reingold algorithm to bring strongly connected nodes closer together [64].

#### Flow Network Construction

To investigate the symptoms associated with SI and their location in the network, we used the *flow* function in the *qgraph* package [59] to calculate and visualize the pathways of influence in the IA-SI-CM-bullying network [65]. SI was designated as the source node, located on the far left of the network. The second-level nodes represent symptoms directly related to SI, whereas the third-level and subsequent nodes represent symptoms indirectly associated with SI. Symptom nodes that are positioned nearer to the SI and have a stronger edge indicate a closer relationship with SI.

#### Centrality and Stability

We first calculated four centrality indexes using the R package *qgraph* to assess the importance of each node [66]: (1) strength (the total weight or number of edges connected by a node to other nodes), (2) betweenness (the number of pairs of nodes that pass through the node on the shortest path between other nodes), (3) closeness (a measure of the average distance between a node and other nodes in the network), and (4) expected influence (EI; the sum of the weights of the edges connected to the node disregarding absolute values). However, considering the presence of negative edges in the network and the instability of betweenness and closeness [67], EI was chosen to report node importance [68]. We also used the R package *networktools* to calculate bridge EI (BEI; the number and strength of edges between a node and nodes from different clusters) [69]. Nodes with high BEI may represent factors that facilitate connections among various symptom groups or mental health conditions [70]. In addition, the predictability of the nodes is calculated using the R package *MGM* and displayed in the form of a pie chart in the outer ring of each node [71]. Nodes with high predictability are considered to be more susceptible to other factors in the network [72].

The reliability and stability of the network were evaluated using the R package *bootnet* [73]. First, we used a nonparametric bootstrap method to estimate the 95% CIs (number of bootstrap samples=1000) for edge weights. A narrower CI indicates greater accuracy of the network. Next, we tested node centrality and edge difference. Finally, the correlation stability coefficient (CSC) was calculated using the case-dropping bootstrap method. The CSC represents the maximum proportion of cases that can be discarded while maintaining at least a 95% probability of correlation with the initial centrality index of 0.7. Generally, a CSC value greater than 0.25 is considered acceptable, whereas 0.5 is preferable [73].

#### Network Comparison

The *NetworkComparisonTest* package was used for 1000 permutations to compare differences between male and female subnetworks, as well as between subnetworks with and without NSSI. Three tests were conducted: (1) the network structure invariance test was conducted to assess whether there was a significant difference in the overall structure of the 2 networks, (2) the global strength invariance test was conducted to evaluate the total strength of all connections in the network, and (3) the edge strength invariance test was conducted to assess whether the weights of corresponding edges in the 2 networks are significantly different [74].

### Ethical Considerations

This study was approved by the ethics committee of Second Xiangya Hospital of Central South University (2021; National Ethics Review K013). Informed consent was obtained online from both the adolescents and their legal guardians before data collection, with support from school personnel. To protect the privacy and confidentiality of the participants, all data were anonymized before analysis. Each participant was assigned a unique study identification number, and all personal information was stored separately from the research data, with access restricted to authorized personnel only. As part of the compensation, participants were provided with free psychological assessments and access to professional mental health support if needed.

## Results

### Descriptive Statistics

The final analysis included a total of 6573 participants with a mean age of 14.62 (SD 1.43) years. Most of the participants reported having siblings (5349/6573, 81.38%) and were of Han ethnicity (6428/6573, 97.79%). Furthermore, the sex distribution was relatively balanced, comprising 51.12% (3360/6573) female and 48.88% (3213/6573) male individuals (Table 1). Approximately one-sixth of the participants (1040/6573, 15.82%) indicated a history of SI. The sociodemographic characteristics and experience of IA, CM, bullying, and NSSI were significantly different between the SI group and the non-SI group (all *P*<.001). Specifically, female participants were more likely to report having experienced SI. People with SI had notably higher scores on the subscales related to IA, CM, and bullying than those without SI, and they had significantly more NSSI events (*P*<.001). The chi-square test indicated no significant difference in ethnicity and the distribution of only children between the SI group and the non-SI group.

**Table 1.** Descriptive statistics of the study samples (N=6573).

	Without SI^a^ (n=5533)	With SI (n=1040)	Total (N=6573)	Chi-square (*df*) or *Z* score values	*P* value
Sex, n (%)				58.76 (1)^b^	<.001
Male	2818 (50.93)	395 (37.98)	3213 (48.88)		
Female	2715 (49.07)	645 (62.02)	3360 (51.12)		
Ethnicity, n (%)				0.24 (1)^b^	.06
Han	5419 (97.94)	1009 (97.02)	6428 (97.79)		
Others	114 (2.06)	31 (2.98)	145 (2.21)		
Only child, n (%)				3.44 (2)^b^	.62
Yes	1036 (18.72)	188 (18.08)	1224 (18.62)		
No	4497 (81.28)	852 (81.92)	5349 (81.38)		
Age (y), mean (SD)	14.59 (1.45)	14.80 (1.33)	14.62 (1.43)	−4.23^c^	<.001
Internet addiction, mean (SD)					
Compulsive internet use	5.22 (2.31)	6.80 (2.25)	5.47 (2.37)	−21.18^c^	<.001
Withdrawal reaction	5.40 (2.30)	6.89 (2.17)	5.63 (2.34)	−20.45^c^	<.001
Tolerance	7.62 (3.00)	9.71 (2.66)	7.95 (3.05)	−21.88^c^	<.001
Interpersonal and health-related problems	8.72 (3.75)	10.8 (3.43)	9.06 (3.78)	−18.54^c^	<.001
Time management	7.04 (2.90)	8.99 (2.76)	7.34 (2.97)	−21.46^c^	<.001
Childhood maltreatment, mean (SD)					
Emotional abuse	6.29 (2.21)	9.81 (4.22)	6.84 (2.93)	−32.14^c^	<.001
Physical abuse	5.54 (1.64)	6.72 (2.99)	5.72 (1.97)	−20.36^c^	<.001
Emotional neglect	9.73 (5.71)	12.73 (5.34)	10.20 (5.76)	−18.79^c^	<.001
Physical neglect	7.79 (2.99)	8.82 (3.19)	7.95 (3.05)	−10.53^c^	<.001
Bullying, mean (SD)					
Traditional bullying victimization	0.11 (0.48)	0.38 (0.82)	0.15 (0.56)	−16.00^c^	<.001
Cyberbullying victimization	0.07 (0.34)	0.26 (0.66)	0.10 (0.44)	−16.02^c^	<.001
Traditional bullying perpetration	0.06 (0.36)	0.14 (0.51)	0.07 (0.39)	−9.46^c^	<.001
Cyberbullying perpetration	0.04 (0.33)	0.10 (0.45)	0.05 (0.35)	−7.46^c^	<.001
NSSI^d^ history, mean (SD)	0.12 (0.55)	1.16 (1.15)	0.28 (0.87)	−34.90^c^	<.001

a SI: suicidal ideation.

b Chi-square (*df*) values.

c *Z* score values.

d NSSI: nonsuicidal self-injury.

### Mediation Model

**Figure 1.**
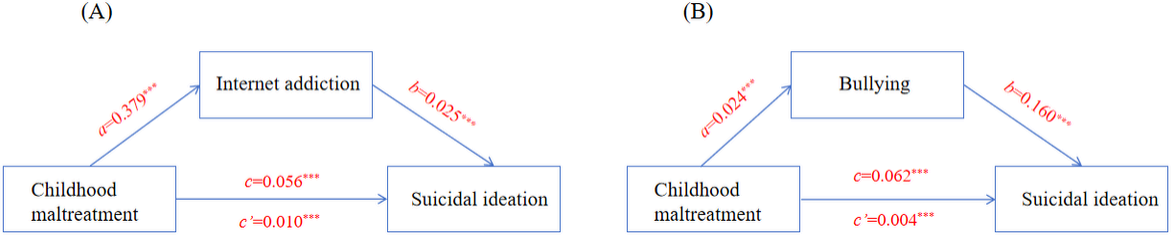
Mediation models of childhood maltreatment and suicidal ideation through internet addiction or bullying. Childhood maltreatment is the independent variable, suicidal ideation is the dependent variable, internet addiction is the mediating variable in (A), and bullying is the mediating variable in (B). ^***^P<.001; *c*=direct effect; *c*’=indirect effect.

As shown in Figure 1, a total of 2 mediation models were established to examine the indirect effects of CM on SI via IA and bullying. In the first model, CM was positively associated with IA (*a*=0.379, 95% CI 0.348-0.410; bootstrap SE 0.016), and IA was positively associated with SI (*b*=0.025, 95% CI 0.022-0.029; bootstrap SE 0.002). The indirect effect of CM on SI via IA was significant (*c’*=0.010, 95% CI 0.008-0.011; bootstrap SE 0.001), whereas the direct effect remained significant (*c*=0.056, 95% CI 0.051-0.061; bootstrap SE 0.002). In the second model, CM was positively associated with bullying (*a*=0.024, 95% CI 0.020-0.027; bootstrap SE 0.002), and bullying was positively associated with SI (*b*=0.160, 95% CI 0.128-0.192; bootstrap SE 0.017). The indirect effect via bullying was also significant (*c’*=0.004, 95% CI 0.002-0.005; bootstrap SE 0.001), with a direct effect of *c*=0.062 (95% CI 0.061-0.070; bootstrap SE 0.002). These results suggest that both IA and bullying partially mediate the association between CM and SI, with IA showing a slightly stronger mediating effect.

### Network Structure

**Figure 2.**
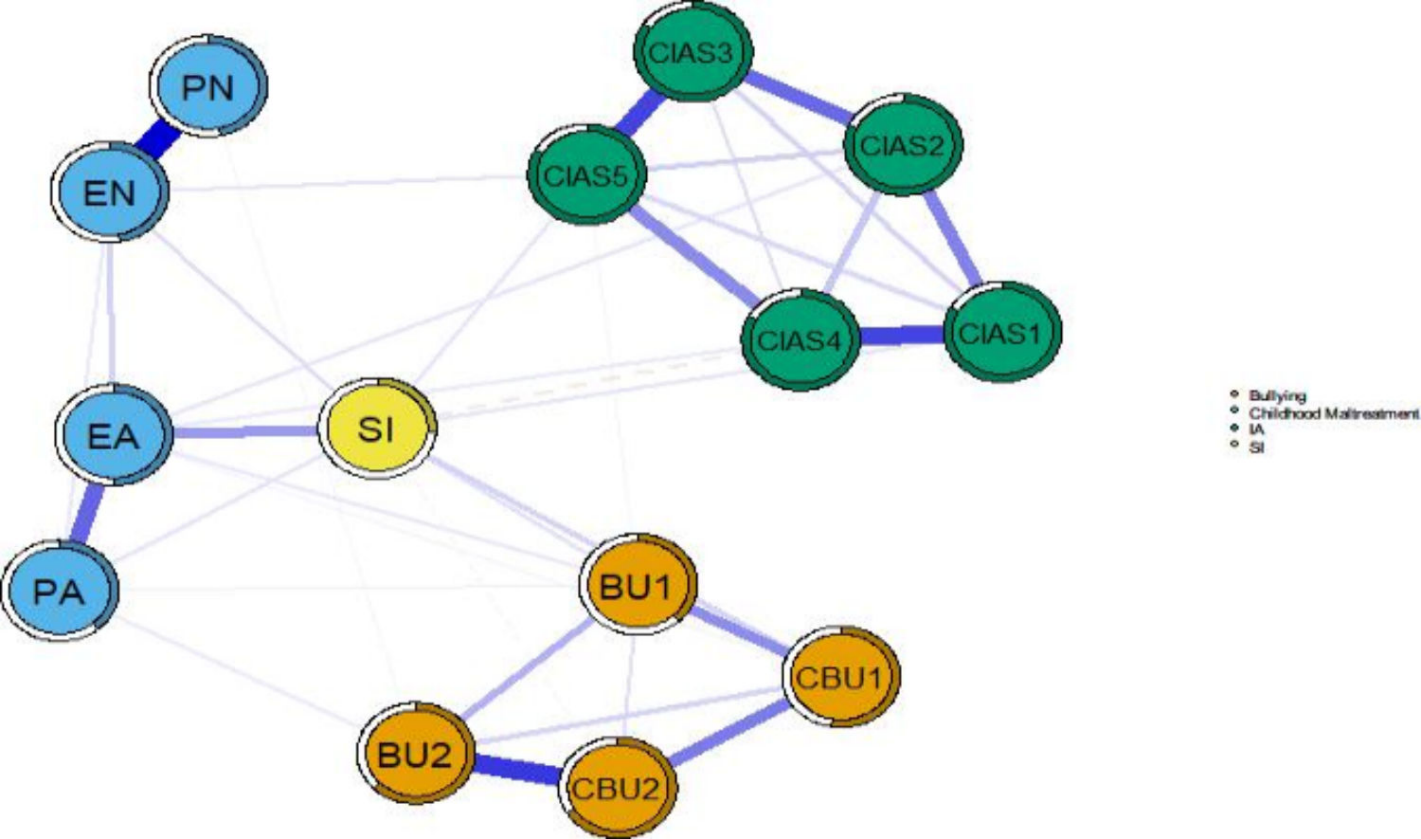
Network structure of suicidal ideation (SI), internet addiction (IA), bullying, and childhood maltreatment (CM) in adolescents. Symptoms within the same symptom cluster are represented by nodes of the same color: orange for bullying, blue for CM, green for IA, and yellow for SI. Blue edges indicate a positive correlation, and red edges indicate a negative correlation. Thicker edges indicate stronger correlations. BU1: traditional bullying victimization; BU2: traditional bullying perpetration; CBU1: cyberbullying victimization; CBU2: cyberbullying perpetration; CIAS1: compulsive internet use; CIAS2: withdrawal reaction; CIAS3: tolerance; CIAS4: interpersonal and health-related problems; CIAS5: time management; EA: emotional abuse; EN: emotional neglect; PA: physical abuse; PN: physical neglect.

The network structure of SI, bullying, IA, and CM is shown in Figure 2. Among the 91 possible edges, 38 (42%) were nonzero (density=41.8%; mean weight 0.065), indicating a high correlation between symptoms. From the visual results, we observed that the strongest edge in the network was between EN and PN, followed by the edge between traditional bullying perpetration and cyberbullying perpetration. These findings are consistent with the results of the nonparametric bootstrap test (Figure S1 in Multimedia Appendix 1). In addition, the SI node was positioned at the center of the entire network, suggesting that SI may play a key role in the interplay among bullying, IA, and CM. It is worth noting that, although there were significant internal correlations within both the IA and bullying symptom groups, the relationship between these 2 groups was minimal, highlighting their relative independence. Moreover, the network nodes exhibited a high level of predictability, with an average predictability of 0.619. The predictability of SI was 0.263, indicating that 26.3% of the variance can be explained by other nodes (Table 2).

**Table 2.** The standardized centrality indexes and predictability of the network node.

Node	Expected influence	Strength	Closeness	Betweenness	Predictability
SI^a^	−1.549	−0.600	2.479	2.300	0.263
BU1^b^	−0.800	–1.011	−1.130	−0.858	0.379
CBU1^c^	–0.115	−0.292	−0.011	1.860	0.528
BU2^d^	−0.087	−0.262	−1.424	−0.858	0.627
CBU2^e^	–0.110	0.172	−0.921	0.106	0.665
EA^f^	0.454	0.305	1.506	0.194	0.500
PA^g^	−1.707	−1.964	0.215	−0.858	0.388
EN^h^	0.440	0.291	0.239	0.194	0.471
PN^i^	−1.309	–1.545	–0.386	−0.858	0.468
CIAS1^j^	1.015	0.894	−0.178	−0.770	0.829
CIAS2^k^	0.777	0.645	−0.258	−0.244	0.817
CIAS3^l^	1.290	1.183	0.184	0.457	0.840
CIAS4^m^	0.571	1.168	−0.146	−0.600	0.826
CIAS5^n^	1.130	1.015	−0.168	−0.069	0.835

a SI: suicidal ideation.

b BU1: traditional bullying victimization.

c CBU1: cyberbullying victimization.

d BU2: traditional bullying perpetration.

e CBU2: cyberbullying perpetration.

f EA: emotional abuse.

g PA: physical abuse.

h EN: emotional neglect.

i PN: physical neglect.

j CIAS1: compulsive internet use.

k CIAS2: withdrawal reaction.

l CIAS3: tolerance.

m CIAS4: interpersonal and health-related problems.

n CIAS5: time management.

**Figure 3.**
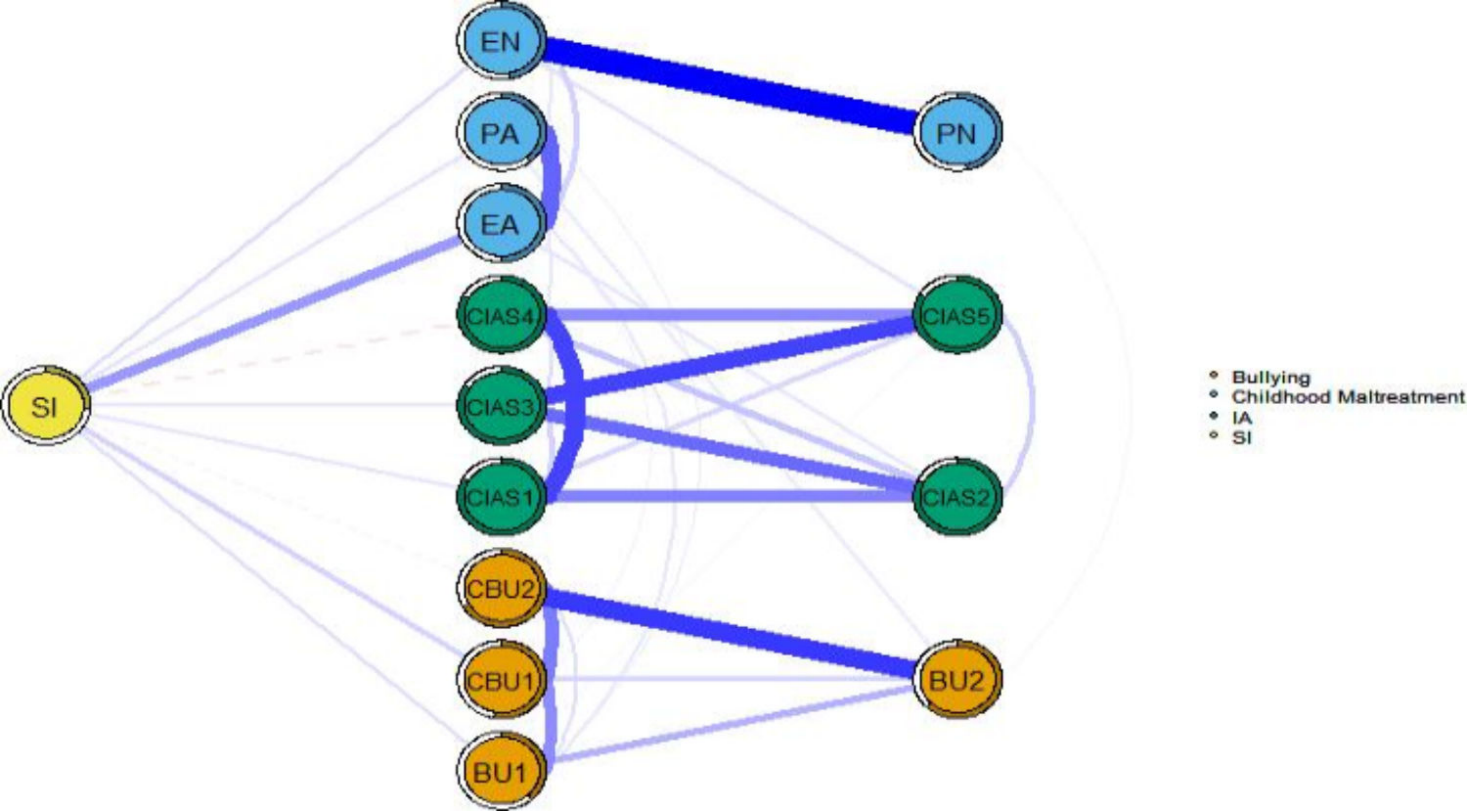
Flow network of suicidal ideation (SI), internet addiction (IA), bullying, and childhood maltreatment (CM) in adolescents. Symptoms within the same symptom cluster are represented by nodes of the same color: orange for bullying, blue for CM, green for IA, and yellow for SI. Blue edges indicate a positive correlation, and red dashed edges indicate a negative correlation. Thicker edges indicate stronger correlations. BU1: traditional bullying victimization; BU2: traditional bullying perpetration; CBU1: cyberbullying victimization; CBU2: cyberbullying perpetration; CIAS1: compulsive internet use; CIAS2: withdrawal reaction; CIAS3: tolerance; CIAS4: interpersonal and health-related problems; CIAS5: time management; EA: emotional abuse; EN: emotional neglect; PA: physical abuse; PN: physical neglect.

Figure 3 shows the flow network of SI, IA, bullying, and CM. All nodes except for PN, time management, withdrawal reaction, and traditional bullying perpetration are directly connected to SI. In addition, all other nodes are associated with SI by no more than 2 intermediate steps. This suggests that SI is a relatively concentrated phenomenon that can be rapidly influenced by other psychological factors or behaviors. The node with the strongest direct positive correlation with SI was EA, followed by cyberbullying victimization. Conversely, interpersonal and health problems and cyberbullying perpetration exhibited the strongest direct negative associations with SI.

### Central and Bridge Symptoms

**Figure 4.**
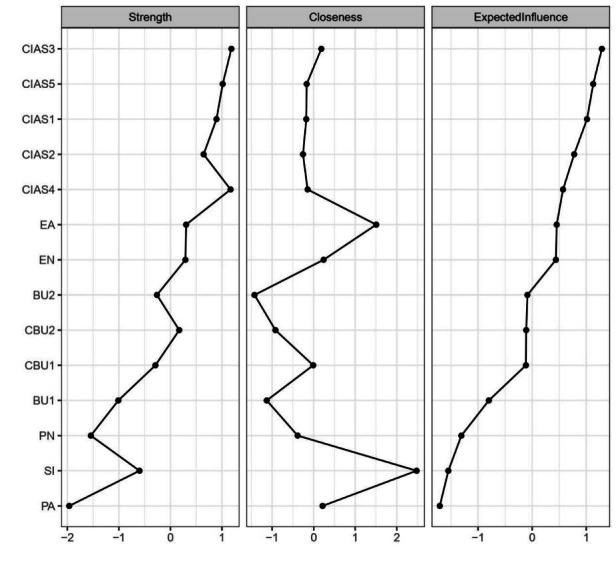
Centrality metrics for each node of the network (standardized values). BU1: traditional bullying victimization; BU2: traditional bullying perpetration; CBU1: cyberbullying victimization; CBU2: cyberbullying perpetration; CIAS1: compulsive internet use; CIAS2: withdrawal reaction; CIAS3: tolerance; CIAS4: interpersonal and health-related problems; CIAS5: time management; EA: emotional abuse; EN: emotional neglect; PA: physical abuse; PN: physical neglect; SI: suicidal ideation. The y-axis shows the node names, and the x-axis shows the standardized values of the centrality indices (strength, closeness, and expected influence).

**Figure 5.**
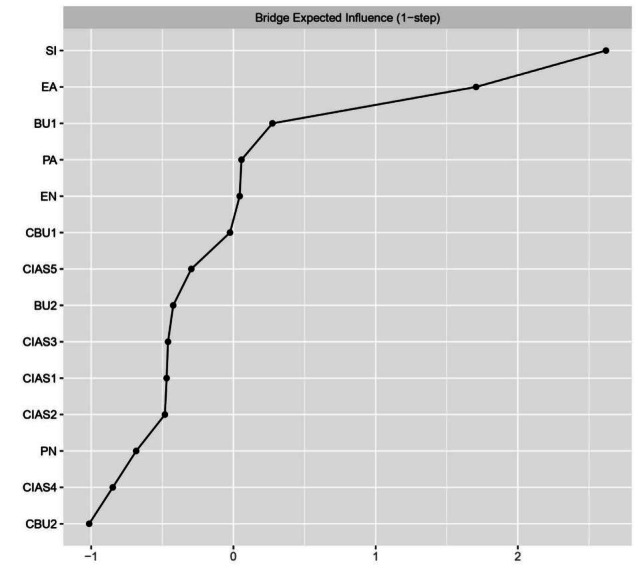
Bridge expected influence for each node of the network (ranked by *z* scores). BU1: traditional bullying victimization; BU2: traditional bullying perpetration; CBU1: cyberbullying victimization; CBU2: cyberbullying perpetration; CIAS1: compulsive internet use; CIAS2: withdrawal reaction; CIAS3: tolerance; CIAS4: interpersonal and health-related problems; CIAS5: time management; EA: emotional abuse; EN: emotional neglect; PA: physical abuse; PN: physical neglect; SI: suicidal ideation. The y-axis shows the node names, and the x-axis shows the standardized values of the bridge expected influence.

The centrality of the IA-SI-CM-bullying network nodes is shown in Table 2 and Figure 4. On the basis of the EI, the core symptoms of the entire network were tolerance (EI=1.290), time management (EI=1.130), and compulsive internet use (EI=1.015), which were aligned with the results of the bootstrap difference test for EI (Figure S2 in Multimedia Appendix 1). Furthermore, the nodes with the highest BEI were SI, EA, and traditional bullying victimization, which means that these 3 symptoms were identified as bridge symptoms within the network and served a crucial mediating function (Figure 5).

### Network Stability and Accuracy

Figure S3 in Multimedia Appendix 1 shows the results of the node drop stability analysis. The CSC for both the EI and BEI nodes was 0.75, indicating the high stability of the model. The 95% CI obtained through the nonparametric bootstrap method was narrow, suggesting that the network model has predictive accuracy and effectively reflects the real relationship of each node in the network (Figure S4 in Multimedia Appendix 1).

### Network Comparison on History of NSSI and Gender

**Figure 6.**
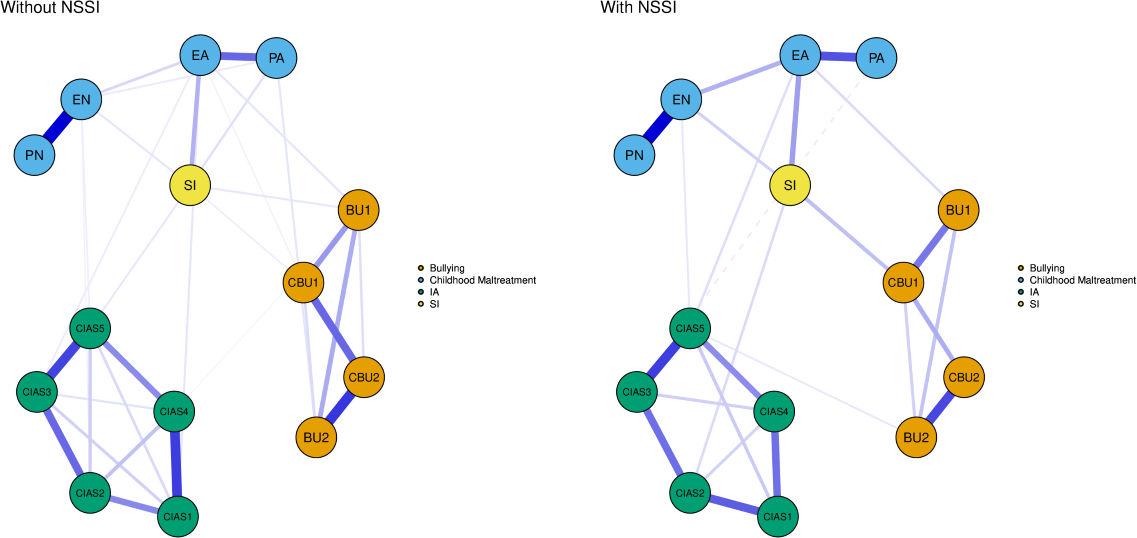
Network structure of suicidal ideation (SI), internet addiction (IA), bullying, and childhood maltreatment (CM) in individuals with or without a history of nonsuicidal self-injury (NSSI). Symptoms within the same symptom cluster are represented by nodes of the same color: orange for bullying, blue for CM, green for IA, and yellow for SI. Blue edges indicate a positive correlation, and red dashed edges indicate a negative correlation. Thicker edges indicate stronger correlations. BU1: traditional bullying victimization; BU2: traditional bullying perpetration; CBU1: cyberbullying victimization; CBU2: cyberbullying perpetration; CIAS1: compulsive internet use; CIAS2: withdrawal reaction; CIAS3: tolerance; CIAS4: interpersonal and health-related problems; CIAS5: time management; EA: emotional abuse; EN: emotional neglect; PA: physical abuse; PN: physical neglect.

The network structure invariance test revealed significant differences between the NSSI and non-NSSI groups (mean 0.268; *P*=.03), suggesting that NSSI significantly influences the associations within the IA-SI-CM-bullying symptom network. In addition, the edge strength invariance test identified significant differences between the 2 networks. Specifically, in individuals with a history of NSSI, stronger relationships were found among EA, EN, cyberbullying victimization, and SI. In contrast, for individuals without a history of NSSI, traditional bullying victimization and interpersonal and health-related problems showed a stronger association with SI (Figure 6). However, no significant differences in global strength were observed between the groups (with NSSI=5.803 vs without NSSI=6.451; *S*=0.648; *P*=.09).

In the gender subgroup analysis, no significant differences were found in global strength (male individuals=6.530 vs female individuals=6.435; *S*=0.095; *P*=.69), but significant differences were observed in the network invariance tests (mean 0.174; *P*=.01; Figure S5 in Multimedia Appendix 1).

## Discussion

### Principal Findings

This study is the is the first to explore the relationships among SI, bullying, CM, and IA in a large sample of Chinese adolescents based on the network model. Two main hypotheses were supported. First, CM showed indirect associations with SI through its links with IA and bullying. Second, network analysis revealed that symptoms of CM, IA, bullying, and SI were positively interconnected. *Tolerance*, *time management*, and *compulsive internet use* emerged as central symptoms, whereas *emotional abuse*, *traditional bullying victimization*, and *suicidal ideation* functioned as bridge symptoms linking distinct domains. EA and cyberbullying victimization showed the strongest associations with SI.

Our findings identified tolerance and time management difficulties as the most central symptoms in the IA-CM-bullying-SI symptom network. Tolerance reflects the need for increasing online engagement to achieve the same satisfaction and often indicates reward dysregulation, whereas poor time management reflects failures in cognitive control and planning [75-78]. These patterns align with self-regulation theory, which posits that goal-directed behavioral control in the face of stress or temptation is crucial for maintaining mental health [79]. Rather than being isolated traits, tolerance and time management difficulties appear to play a functional role in sustaining the broader network of psychosocial adversity and SI. This is consistent with our mediation findings, suggesting that adolescents exposed to adverse events may turn to the internet as a maladaptive coping strategy, reinforcing compulsive use, time mismanagement, reduced real-world interactions, and increased emotional isolation [80]. This reinforcing loop may heighten vulnerability to SI, especially among adolescents facing elevated stress and limited offline support [75,80,81]. Specifically, this pattern may be especially relevant during adolescence, which is a developmental window marked by ongoing maturation of self-regulation systems. As such, deficits in tolerance and time management may demonstrate disproportionately strong associations with mental health outcomes such as SI during adolescence [82]. Encouragingly, the centrality of these symptoms also suggests their potential as early, modifiable targets for transdiagnostic intervention. Brief cognitive behavioral or skill-based programs focused on improving self-regulation may help disrupt this maladaptive network and reduce suicide risk in adolescents.

In the network, SI emerged as the strongest bridge symptom, underscoring its pivotal role in linking IA, CM, and bullying. This pattern highlights SI’s transdiagnostic nature and its sensitivity to a broad range of psychosocial stressors. Consistent with ecological systems theory, which conceptualizes individuals’ development as shaped by nested environmental influences, SI reflects the convergence of psychosocial stressors across family (CM), peer (bullying), and digital (eg, IA) domains [83-85]. Our study further revealed that EA and cyberbullying victimization exhibited the strongest correlations with SI among adolescents. This finding is particularly important in the current social context, where increasing internet use and evolving family dynamics may reshape risk factors for SI [86,87]. Cyberbullying represents a novel and increasingly pervasive form of peer victimization, whereas the strong association between EA and SI may reflect growing emotional dependence on family support. This suggests that emotional forms of harm, especially in close relationships, may have a growing impact on youth mental health [88,89]. From the perspective of the interpersonal theory of suicide, SI arises from thwarted belongingness, perceived burdensomeness, and the capacity for self-harm [90]. EA and cyberbullying may contribute to SI by amplifying perceived burdensomeness and thwarted belongingness. Adolescents exposed to EA may experience heightened isolation and unwantedness, exacerbating perceptions of thwarted belongingness and burdensomeness, which, in turn, intensify SI [91]. Similarly, the inescapable nature of cyberbullying due to its anonymity and constant digital exposure may deprive victims of safe social spaces, increasing psychological distress and social and emotional disconnection [86,87,92,93]. This finding reinforces the critical importance of addressing SI in adolescence and highlights the necessity of this research. It also suggests that intervention priorities must evolve with the times, with increasing attention to cyberbullying and EA within the family as key targets for prevention efforts.

Distinct network patterns emerged based on NSSI history. Among individuals with a history of NSSI, stronger associations were observed between SI and EA, EN, and cyberbullying victimization, reflecting heightened vulnerability to interpersonal trauma [94]. This heightened vulnerability can be attributed to maladaptive emotional regulation strategies, which are frequently observed in individuals with a history of NSSI. Studies have shown that heightened emotional reactivity and increased avoidance behaviors often characterize these adolescents, making them less equipped to cope with distressing experiences [94,95]. Furthermore, NSSI may act as a maladaptive mechanism to manage intense negative emotions or suicidal impulses [95,96]. EA and EN foster chronic emotional dysregulation, which, when combined with history such as NSSI, amplifies feelings of helplessness, rejection, and distress, increasing susceptibility to SI [97,98]. Conversely, for individuals without a history of NSSI, SI was more closely linked to traditional bullying victimization and interpersonal or health-related problems, suggesting that situational stressors rather than chronic trauma play a greater role in this group. Traditional bullying victimization may act as a catalyst for SI by inducing negative emotion, social isolation, and hopelessness [99]. Similarly, health-related problems and interpersonal difficulties may create substantial stress that contributes to the development of SI even in the absence of NSSI [100]. This suggests a divergence in the underlying mechanisms driving SI, with individuals with a history of NSSI showing heightened sensitivity to chronic emotional trauma and individuals without a history of NSSI being more affected by external, situational stressors. Interventions should be tailored to these distinct pathways, addressing emotional regulation deficits in individuals with a history of NSSI and external stressors in others.

### Implications

These findings emphasize the importance of multilevel and tailored interventions to reduce suicide risk. Prevention efforts should prioritize addressing CM, bullying victimization, and time management difficulties in schools and other environments. For individuals with a history of NSSI, therapeutic approaches should focus on mitigating the long-term effects of emotional trauma by enhancing emotion regulation skills. For those without NSSI histories, interventions should target situational stressors such as bullying and interpersonal challenges, with strategies to strengthen social support and adaptive coping mechanisms. An integrated approach combining school-based antibullying programs, time management training, and family-centered interventions addressing emotional maltreatment could create a comprehensive framework for reducing suicide risk and improving adolescent mental health. Importantly, these implications should be interpreted within the cultural context of China, where high parental control, limited emotional expressiveness in families, and the widespread use of digital platforms for peer interaction may uniquely influence adolescents’ psychosocial stressors [101,102]. While the key factors identified, such as EA, cyberbullying, and internet exposure, have been observed across different societies [91,103,104], cross-cultural validation is needed to confirm their generalizability and guide culturally tailored interventions.

### Limitations

Several limitations should be noted. First, the cross-sectional design precludes conclusions about causal or temporal relationships between SI and other symptoms, highlighting the need for longitudinal studies to clarify these dynamics. Second, reliance on self-reported scales may introduce bias due to subjective perceptions or recall inaccuracies. Future research should incorporate clinician-administered assessments or objective measures to enhance reliability. Third, the sample may not fully represent the broader adolescent population due to potential demographic or cultural biases, limiting generalizability. Expanding future studies to more diverse samples would improve external validity and deepen understanding of these relationships across different contexts. Fourth, this study did not include depression and anxiety in the analyses, which may have led to overestimated associations or overlooked potential effects among variables. Future research should consider incorporating these emotional factors and applying more comprehensive models to better capture the relationships.

### Conclusions

This study used mediation and network models to examine the relationships among SI, IA, bullying, and CM. Tolerance and time management emerged as central symptoms, whereas SI was the strongest bridge symptom. EA and cyberbullying victimization had the strongest associations with SI. Notably, significant differences in network structure were observed between adolescents with and without a history of NSSI, with individuals with a history of NSSI showing stronger links to emotional trauma and individuals without a history of NSSI being more affected by situational stressors. These findings highlight the need for tailored, multilevel interventions addressing CM, bullying, and IA symptoms to improve adolescent mental health and reduce suicide risk.

## Supplementary material

10.2196/79858 Multimedia Appendix 1 Network weight matrices, results of network accuracy and difference tests, network structures for male and female individuals, and centrality measures across subgroups defined by sex and nonsuicidal self-injury (NSSI) status.

## Abbreviations

BEI: bridge expected influence
CM: childhood maltreatment
CSC: correlation stability coefficient
EA: emotional abuse
EI: expected influence
EN: emotional neglect
IA: internet addiction
NSSI: nonsuicidal self-injury
PN: physical neglect
SI: suicidal ideation
