# Supplementary material for: Childhood Maltreatment, Bullying, and Internet Addiction in Relation to Suicidal Ideation Among Adolescents: Cross-Sectional Mediation and Network Analysis
Source: J Med Internet Res. 2025 Oct 7;27:e79858. doi: 10.2196/79858 (PMC12503435; doi:10.2196/79858)

Table S1. IA-SI-CM-Bullying network node correlation weight matrix

Figure S1. Non-parametric bootstrap difference test for edge difference

Figure S2. Non-parametric bootstrap difference test for EI

Figure S3. The CS-C of expected influence and bridge expected influence

Figure S4. 95% confidence interval (CI) of the edge weights

Figure S5. Network structure of Suicial ideation (SI), Internet addiction (IA), Bullying, and Childhood maltreatment (CM) in males and females

Figure S6. Standardized estimates of male and female network centrality

Figure S7. Standardized estimates of in individuals with or without NSSI network centrality

Table S1 IA-SI-CM-Bullying network node correlation weight matrix

	BU1	CBU1	BU2	CBU2	SI	CIAS1	CIAS2	CIAS3	CIAS4	CIAS5	EA	PA	EN
BU1	1.00	0.79	0.78	0.79	0.40	0.18	0.19	0.19	0.18	0.19	0.29	0.24	0.12
CBU1	0.79	1.00	0.82	0.89	0.42	0.19	0.19	0.20	0.17	0.19	0.31	0.24	0.12
BU2	0.78	0.82	1.00	0.94	0.29	0.14	0.16	0.14	0.15	0.16	0.23	0.23	0.11
CBU2	0.79	0.89	0.94	1.00	-0.27	0.13	0.14	0.14	0.12	0.14	0.20	0.18	0.06
SI	0.40	0.42	0.29	-0.27	1.00	0.33	0.32	0.36	-0.28	0.33	0.49	0.33	0.27
CIAS1	0.18	0.19	0.14	0.13	0.33	1.00	0.86	0.83	0.88	0.83	0.25	0.10	0.22
CIAS2	0.19	0.19	0.16	0.14	0.32	0.86	1.00	0.86	0.84	0.83	0.26	0.11	0.23
CIAS3	0.19	0.20	0.14	0.14	0.36	0.83	0.86	1.00	0.83	0.88	0.25	0.09	0.21
CIAS4	0.18	0.17	0.15	0.12	-0.28	0.88	0.84	0.83	1.00	0.85	0.22	0.10	0.22
CIAS5	0.19	0.19	0.16	0.14	0.33	0.83	0.83	0.88	0.85	1.00	0.25	0.12	0.25
EA	0.29	0.31	0.23	0.20	0.49	0.25	0.26	0.25	0.22	0.25	1.00	0.60	0.24
PA	0.24	0.24	0.23	0.18	0.33	0.10	0.11	0.09	0.10	0.12	0.60	1.00	0.14
EN	0.12	0.12	0.11	0.06	0.27	0.22	0.23	0.21	0.22	0.25	0.24	0.14	1.00
PN	0.15	0.16	0.17	0.17	0.18	0.17	0.17	0.15	0.16	0.20	0.26	0.24	0.66

Figure S1. Non-parametric bootstrap difference test for edge difference

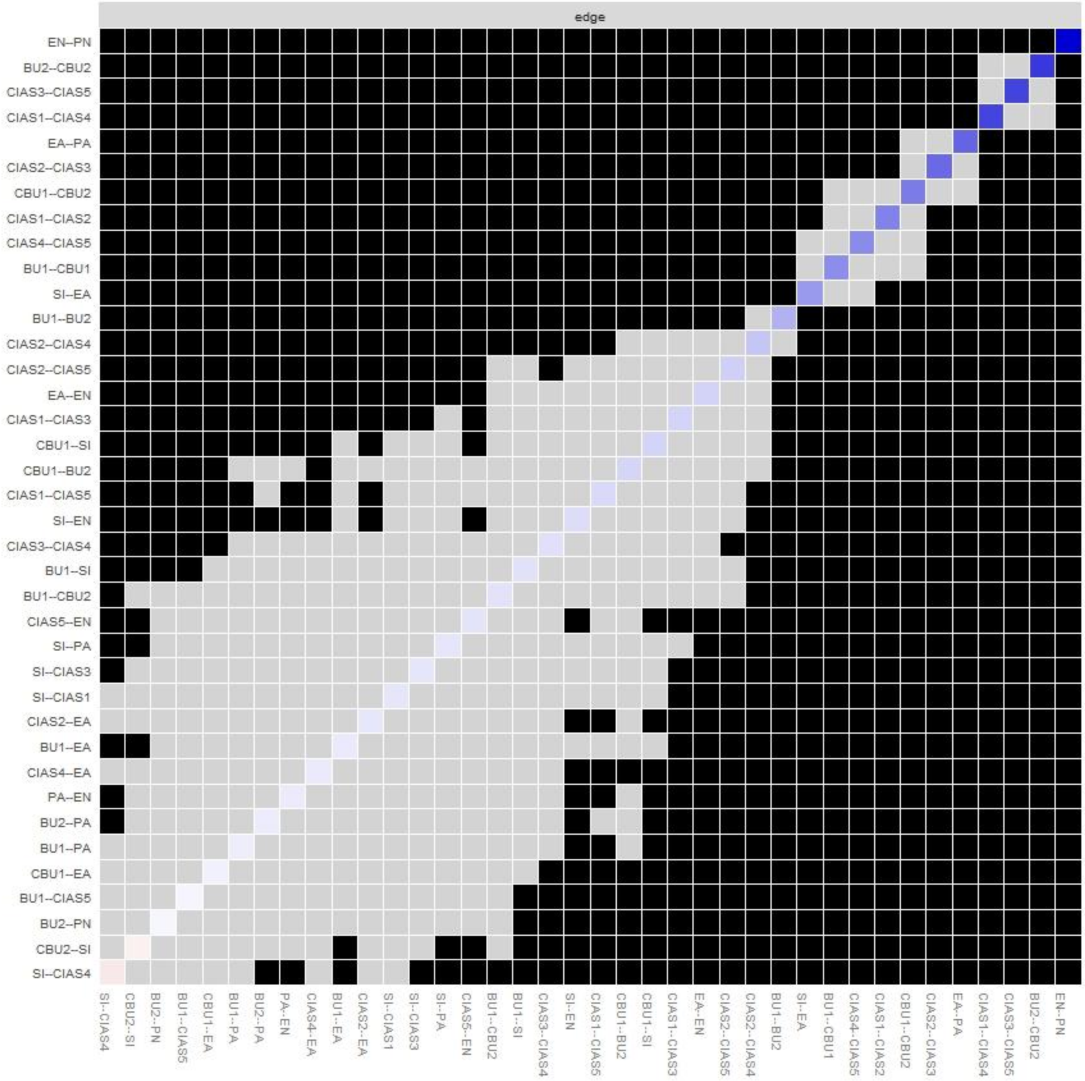

Note: Gray boxes signify edges that exhibit no significant difference from each other, while black boxes indicate edges that show a significant difference from each other at a significance level of α = 0.05.

Figure S2. Non-parametric bootstrap difference test for EI

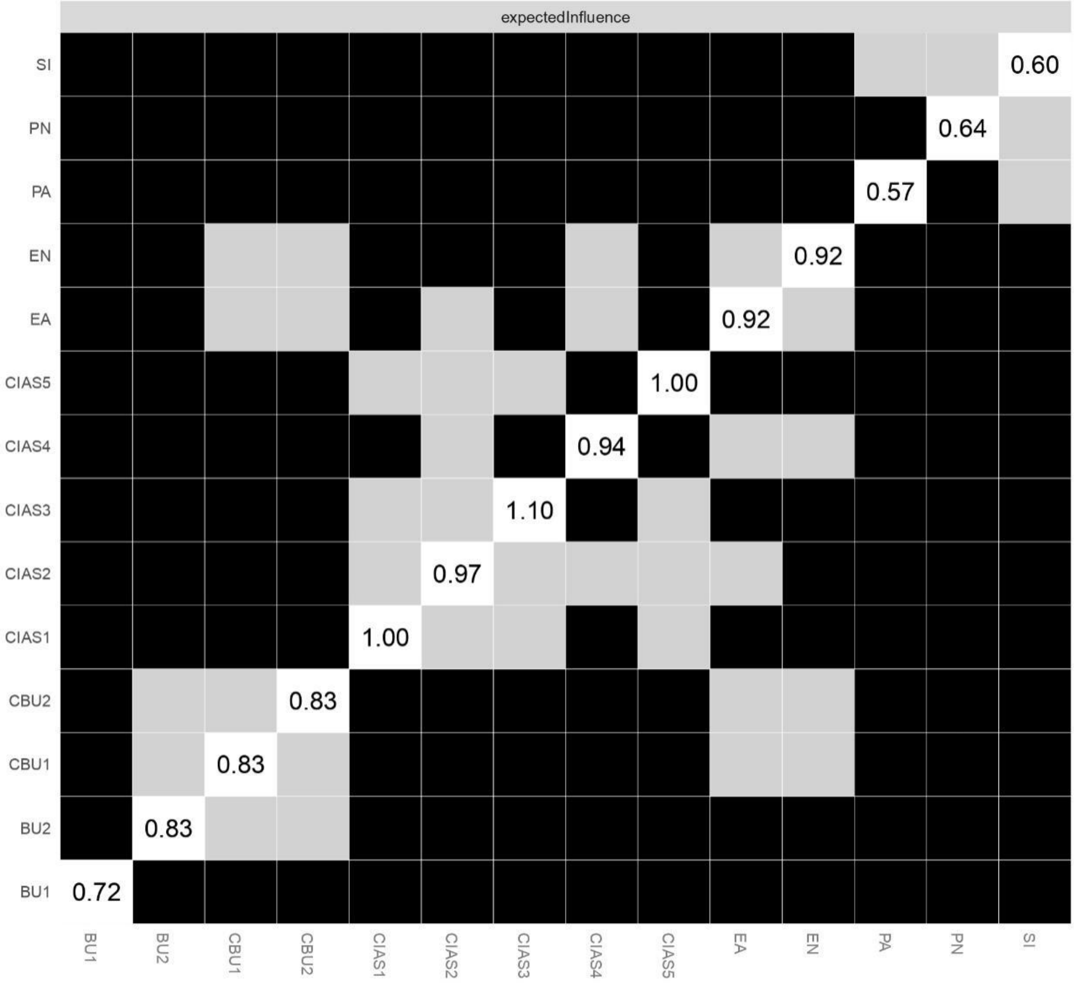

Note: Gray boxes signify edges that exhibit no significant difference from each other, while black boxes indicate edges that show a significant difference from each other at a significance level of α = 0.05.

Figure S3. The CS-C of expected influence and bridge expected influence

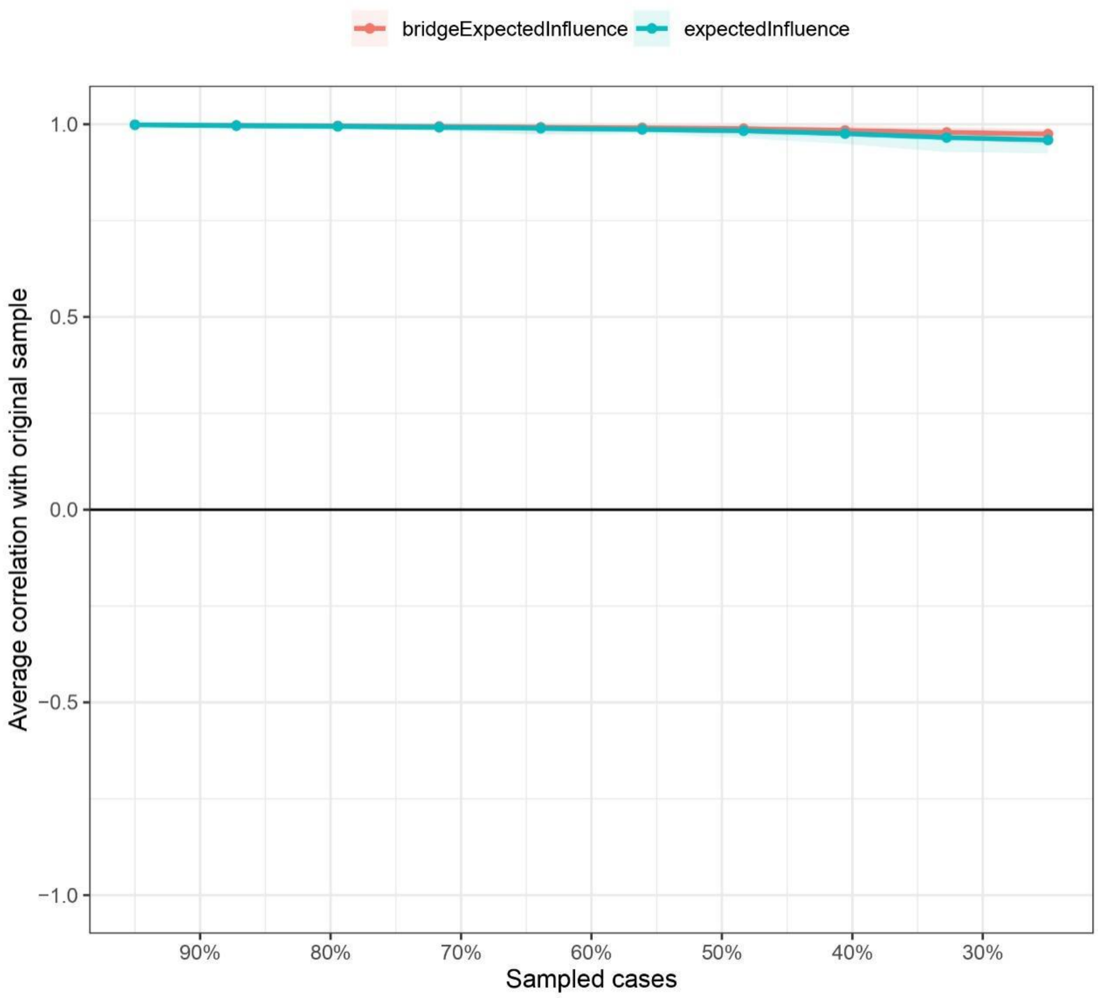

Figure S4. 95% confidence interval (CI) of the edge weights

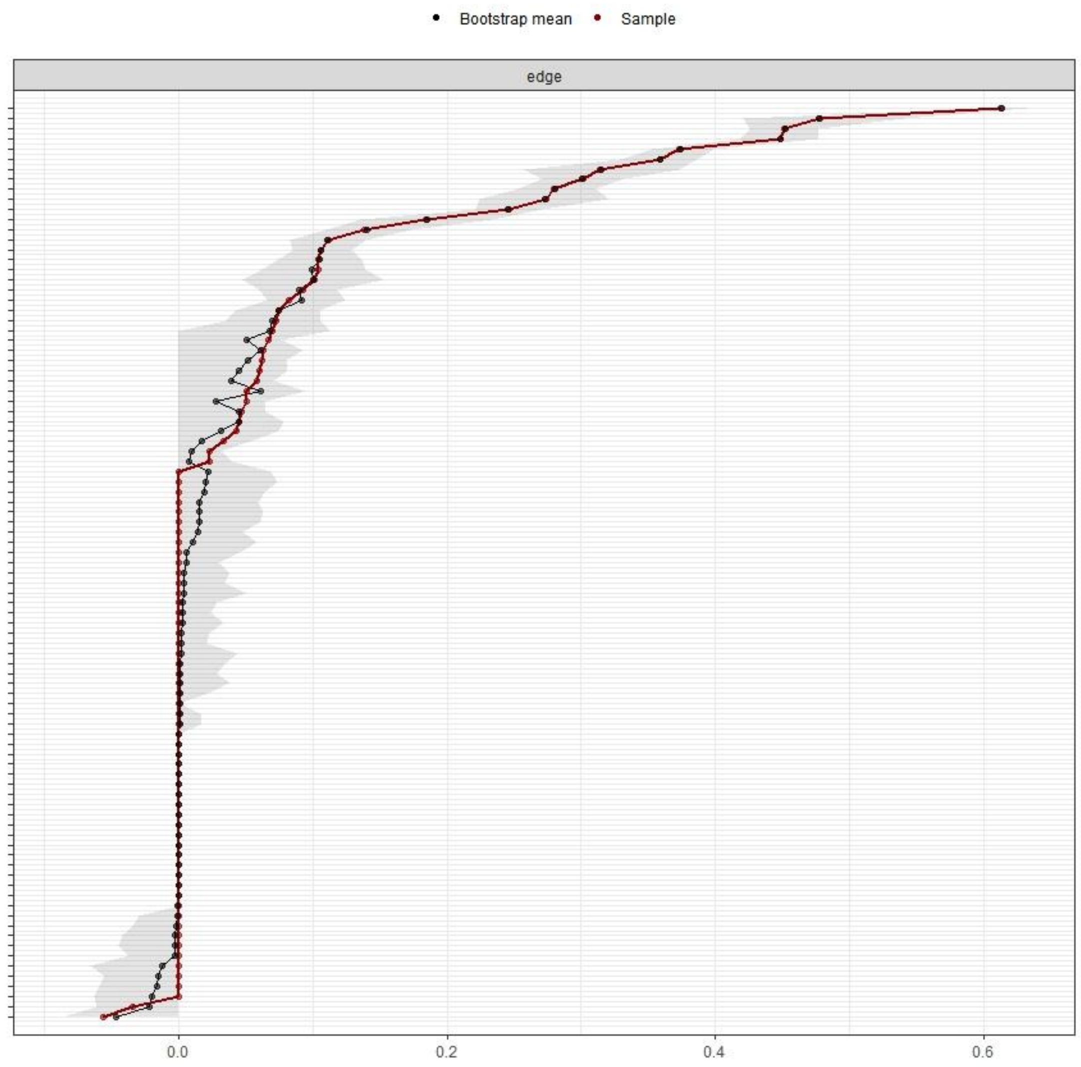

Figure S5. Network structure of Suicial ideation (SI), Internet addiction (IA), Bullying, and Childhood maltreatment (CM) in males and females

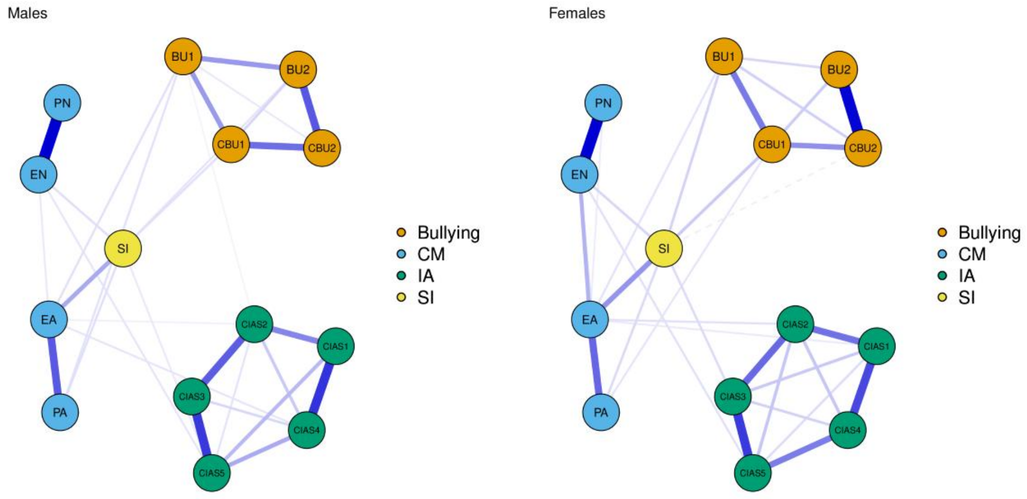

Note: Symptoms within the same symptom cluster are represented by nodes of the same color: orange for bullying, blue for CM, green for IA, and yellow for SI. Blue edges indicate positive correlation and red edges indicate negative correlation. Thicker edges indicate stronger correlations.

Figure S6. Standardized estimates of male and female network centrality

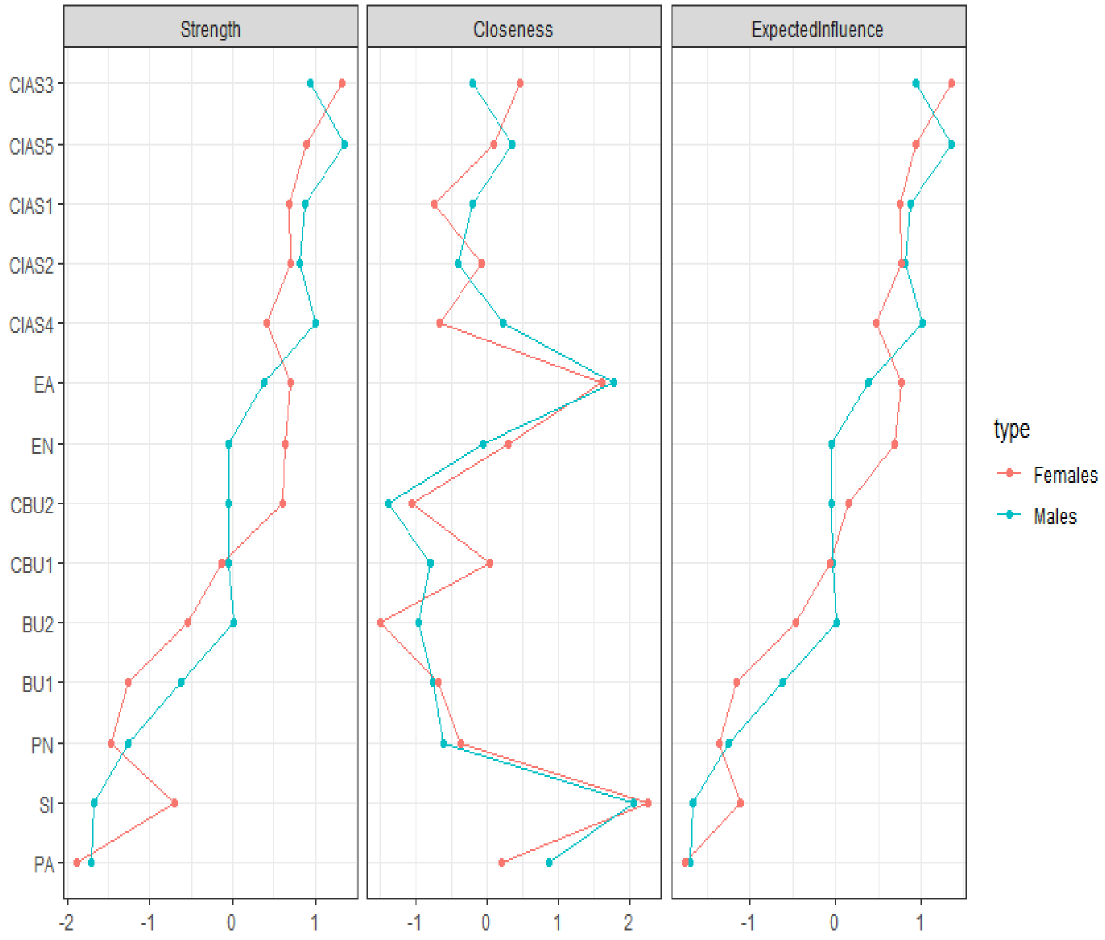

Figure S7. Standardized estimates of individuals with or without NSSI network centrality

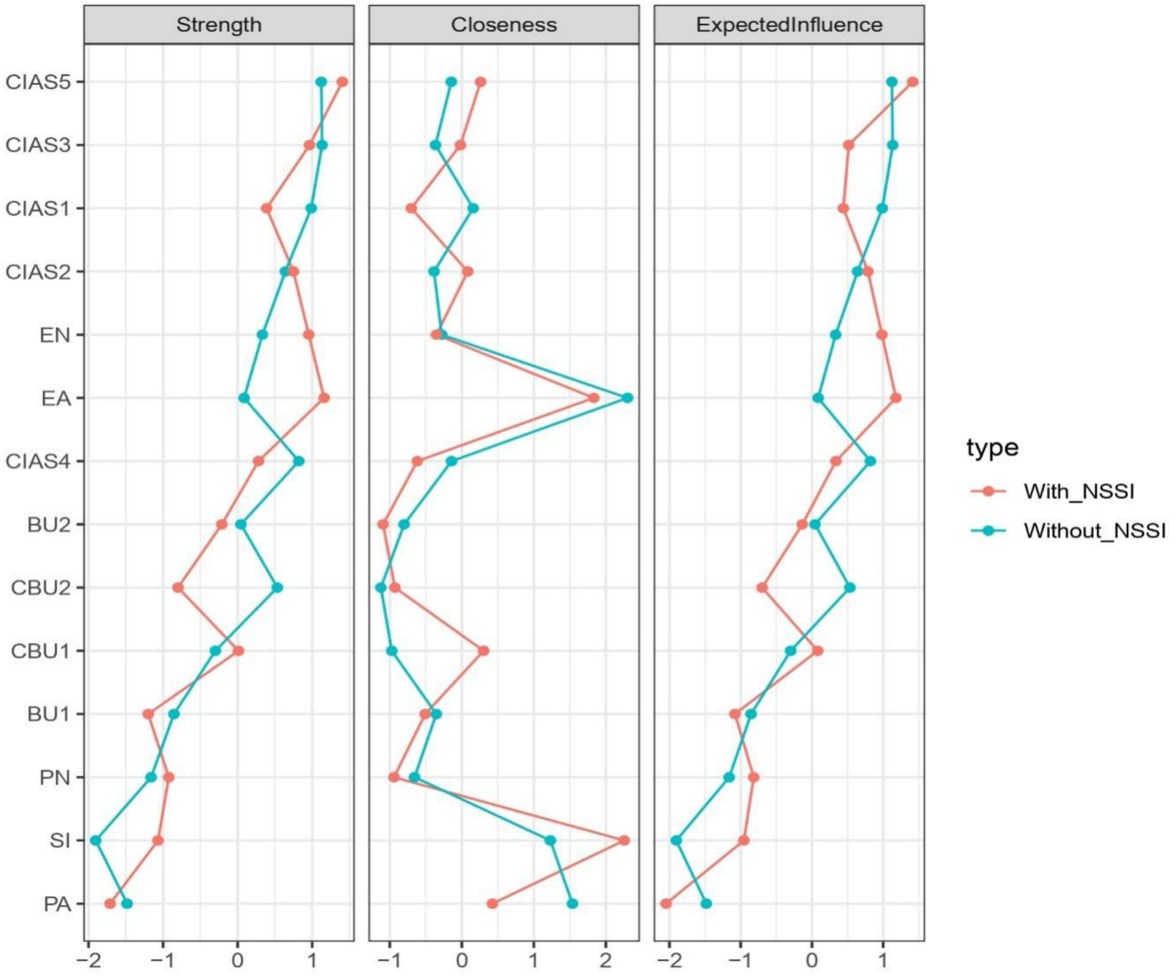

Supplement: Multimedia Appendix 1 [file jmir-v27-e79858-s001.pdf]
